# Evaluation of the selective toxic effect of the charge switchable diethyldithiocarbamate-loaded nanoparticles between hepatic normal and cancerous cells

**DOI:** 10.1038/s41598-018-22915-4

**Published:** 2018-03-15

**Authors:** Marwa M. Abu-Serie

**Affiliations:** Medical Biotechnology Department, Genetic Engineering and Biotechnology Research Institute, City for Scientific Research and Technology Applications (SRTA-City), New Borg EL-Arab, 21934 Alexandria, Egypt

## Abstract

Liver cancer is mainly originated by cancer stem cells (CSCs). Due to difference in pH between normal and tumor cell microenvironments, targeting hepatic CSCs exploiting pH-dependent charge switchable nanoparticles (NPs) is extremely required to limit nonselective toxicity to normal hepatocytes (NHCs) and to completely eliminate the root of cancer origin. In this study, NPs were prepared from cationic chitosan and then coated with anionic albumin namely uncoated and coated NPs, respectively. Both NPs were loaded with diethyldithiocarbamate (DDC) which is an inhibitor of the critical enzyme, aldehyde dehydrogenase (ALDH) 1A1, for CSCs survival. The charge switchable of coated DDC-loaded NPs in neutral and acidic pH (−19 and +28.5 mv, respectively) was illustrated. This special privilege of coated NPs mediated DDC releasing in a slightly acidic pH (tumor microenvironment) rather than a neutral pH (microenvironment of normal cells). Thence, these coated NPs showed the highest selective apoptosis-mediated toxicity only in murine hepatoma cells (Hepa) that may attribute to suppression of NF-κB expression and ALDH1A1 activity, subsequently collapsing 89.7% CD133^+^CSCs. These new findings declare that coated NPs could be promising safe selective anticancer drug for targeting hepatic CSCs and that requires additional future investigations using animal models of liver cancer.

## Introduction

Globally hepatocellular carcinoma (primary liver cancer, HCC) is the third cause of cancer mortality and the fifth most common cancer, affecting over half million individuals per year^1,2^. HCC is mainly derived from rare cancer stem cells (CSCs) pool which originates from normal stem cells/progenitor cells in consequence of mutations. CSCs are capable of uncontrolled self-renewal and metastasis, chemoresistance and tumor recurrence. CSCs possess the critical survival mechanisms and properties crucial for the maintenance and propagation of the tumor^3–5^.

These unique features of hepatic CSCs may be attributed to high aldehyde dehydrogenase (ALDH) 1A1 activity^6^. ALDH1A1 defenses against aldehydes-caused oxidative stress. ALDH1 is considered a general marker for CSC stemness owing to its important role in CSC biology, function, regulation of differentiation and drug resistance^6–8^. ALDH1A1 when coexpressed with CD133, can more specifically characterize hepatic CSC cell population^6^. Thus cytosolic ALDH has been proposed as potential pharmacological targets for treatment of HCC. ALDH is irreversibly inhibited by disulfiram metabolite; diethyldithiocarbamate (DDC) which is produced by hepatic thiol methyltransferases. The subsequent hepatic P450-catalyzed oxidation of DDC metabolite to DDC-sulfoxide and S-methyl-N,N-diethylthiocarbamate (DTC)-sulfoxide and DTC-sulfone which are also an irreversible inhibitor of ALDH1A1^9,10^. The latter DDC metabolite is the most potent ALDH inhibitor^10^. Another factor, NF-κB, has been found to be constitutively expressed in CSCs to maintain stemness by sustaining the undifferentiated and self-renewal states. So, the potency of DDC to suppress NF-κB may be served as a therapeutic target for cancer by collapsing CSCs population^11^.

However, DDC has diverse applications as an agricultural pesticide and as a pharmacological agent against *Mycobacterium tuberculosis*, acquired immunodeficiency syndrome and cutaneous leishmaniasis^12–14^. It displayed adverse effects that mainly related to its metal-chelating ability (copper, iron and zinc) and high affinity for sulfhydryl group-containing proteins^15,16^. Previous studies proved *in vitro* cytotoxicity of DDC against normal Chinese hamster lung fibroblasts V79 cells and zebrafish that were used as model systems for cytotoxicity studies^17,18^. Moreover, its utility had relatively high toxicity *in vivo* including vasoconstriction^19^, hepatotoxicity^20^ and peripheral neurotoxicity which is characterized by myelin injury^21^.

Using nanoparticles (NPs) in cancer therapy is anticipated to overcome the existing obstacles of anticancer drugs by increasing their stability, sustaining their release and decreasing their toxicity against healthy tissues. Due to the controlled size of NPs, it allows anticancer drugs to be delivered selectively to cancer cells and interact with intracellular biomolecules causing targeting of cancer death with low toxic effects on normal cells^22–24^. On the other hand, physiological conditions (hyperpermeability and acidic microenvironment) of cancer cells provide many benefits to NPs to effectively target them^25,26^. NPs that are based on the cationic polysaccharides chitosan have been promising due to their biodegradability, biocompatibility and non-antigenicity^24^. Its positive charge enhances intracellular uptake of the loaded drugs due to its affinity to negatively charged cell membrane of either normal or cancer cells. Therefore, coating chitosan with negatively charged albumin is highly needed^27^.

Delivery systems based on albumin NPs disclose several advantages such as stability, nontoxicity, high binding capacity for both hydrophobic and hydrophilic drugs and easy surface modification. There are several albumin-bound drug delivery system was applied for cancer therapy such as Abraxane® with consideration of its commercial success. Ovalbumin, bovine serum albumin (BSA) and human serum albumin (HSA) can be used and due to the distinct importance of HSA in several studies, it can be replaced with BSA. Albumin shows stability in pH range of 4–9 and at 60 °C for 10 h^28–31^.

The main scope of this study is a design of more potent anticancer drug targeting CSCs (origin of cancer) but with less toxicity to normal cells. Thus, DDC was loaded into uncoated and albumin-coated chitosan NPs and then their selective toxicity between black mice C57 hepatoma (Hepa 1–6) cell line (Hepa) and black mice C57 normal hepatocytes (NHCs) was examined.

## Results

### Characterization of the prepared uncoated and coated NPs

**Figure 1:**
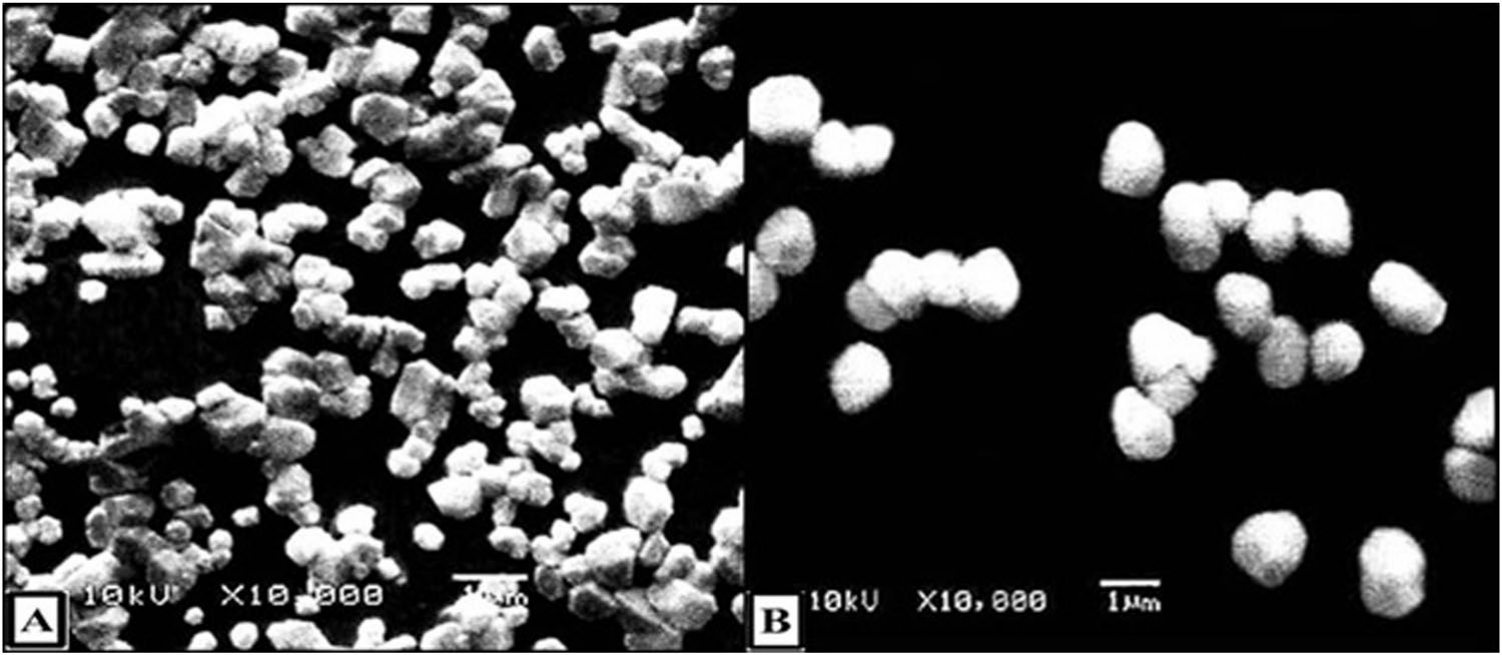
Scanning electron microscope micrographs. (**A)** DDC-loaded NPs (uncoated NPs) and (**B)** DDC-loaded BSA-coated NPs (coated NPs).

Both prepared NPs possessed high loading (71.3%) and encapsulation (95.01%) capacities for DDC that may be attributed to the ionic affinity between negatively charged DDC and positively charged chitosan. These synthetic NPs had appropriate sizes equivalent to 271.9 ± 10.2 and 505 ± 86 nm for uncoated and coated NPs, respectively. The morphology of both NPs coated NPs was illustrated by SEM images (Fig. 1A,B). The zeta potentials of uncoated and coated NPs were +45.4 mv and −19 mv, respectively, considering strongly cationic and anionic charged particles, respectively. After overnight incubation of uncoated NPs and coated NPs in slightly acidic PBS, their charges were +45.4 mv and +28.5 mv, respectively.

### The pH-dependent DDC release from the studied NPs

**Figure 2:**
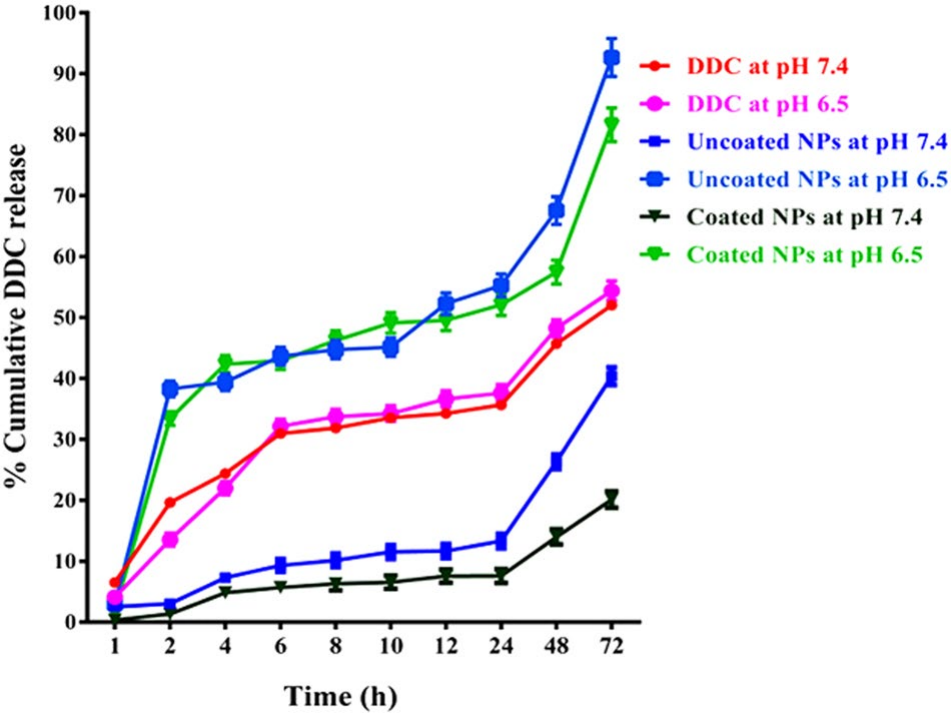
Release profile of DDC from uncoated and coated NPs for 72 h at pH 7.4 and pH 6.5 in comparison with free drug.

Figure 2 demonstrates the slowest release (20.1 ± 1.35%) of DDC from coated NPs at neutral pH through 72 h whereas 81.6 ± 2.8% of DDC was released from coated NPs in acidic pH. This indicates coated NPs decrease liberation of DDC in slightly acidic microenvironment of cancer cells by 4 folds compared to a neutral microenvironment of normal cells. On the other hand, no significant difference was recorded in the release of DDC (>50%) as free drug between pH 7.4 and pH 6.5 during 72 h. Meanwhile, uncoated NPs released 92.7 ± 3.1% and 40.4 ± 1.42% of DDC in pH 6.5 and pH 7, respectively (Fig. 2).

### The selective cytotoxicity of the studied NPs between NHCs and Hepa

**Figure 3:**
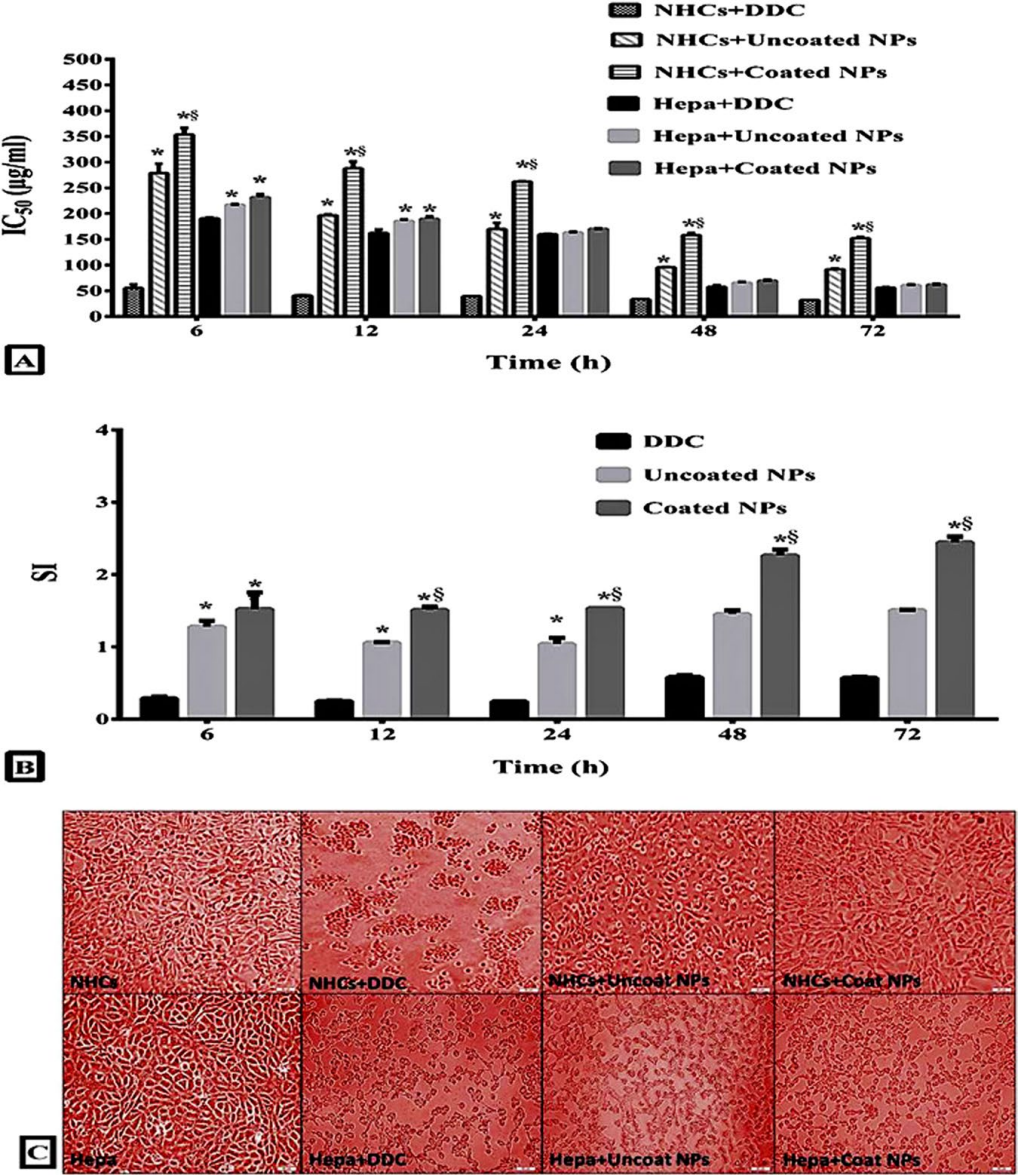
Cytotoxicity of DDC and its NPs to NHCs and Hepa. (**A)** The estimated IC_50_ dose (μg/ml) of DDC, uncoated NPs and coated NPs for inhibiting the growth of NHCs and Hepa for 72 h. (**B)** The calculated SI values of DDC, uncoated NPs and coated NPs between NHCs and Hepa for 72 h (* and ^§^represent significance at p < 0.01 when compared to DDC- and uncoated NPs-treated cells, respectively, at a certain time). (**C)** Morphological alterations of NHCs and Hepa after 72 h treatment with DDC, uncoated NPs and coated NPs. (Magnification x200).

The prepared coated NPs had the lowest significant toxicity against NHCs with high IC_50_ compared to the uncoated NPs (>91 μg/ml and >150 μg/ml, respectively) throughout 72 h compared to IC_50_ of free drug DDC which was less than 55.1 μg/ml. Otherwise, no significant difference was registered between IC_50_ values (55.7 ± 1.2, 60.85 ± 1.1 and 62.82 ± 0.9 μg/ml) of DDC, uncoated NPs and coated NPs, respectively for inhibition growth of Hepa after 72 h incubation (Fig. 3A). Subsequently, coated DDC-loaded NPs exhibited the highest significant selectivity index (SI = 2.5) between normal and cancer liver cells but DDC lack this selectivity (Fig. 3B) as it was illustrated by severe morphological alterations in DDC-treated normal and cancerous liver cells (Fig. 3C). The coated NPs increased the selectivity of DDC by five folds comparably (Fig. 3B). The selective toxicity of uncoated NPs and particularly coated NPs was clarified by the intensive damage in Hepa without any obvious changes in morphology of normal liver cells (Fig. 3C).

### The elective inhibitory potential of the studied NPs on NF-κB expression and ALDH1A1 activity in Hepa

**Figure 4:**
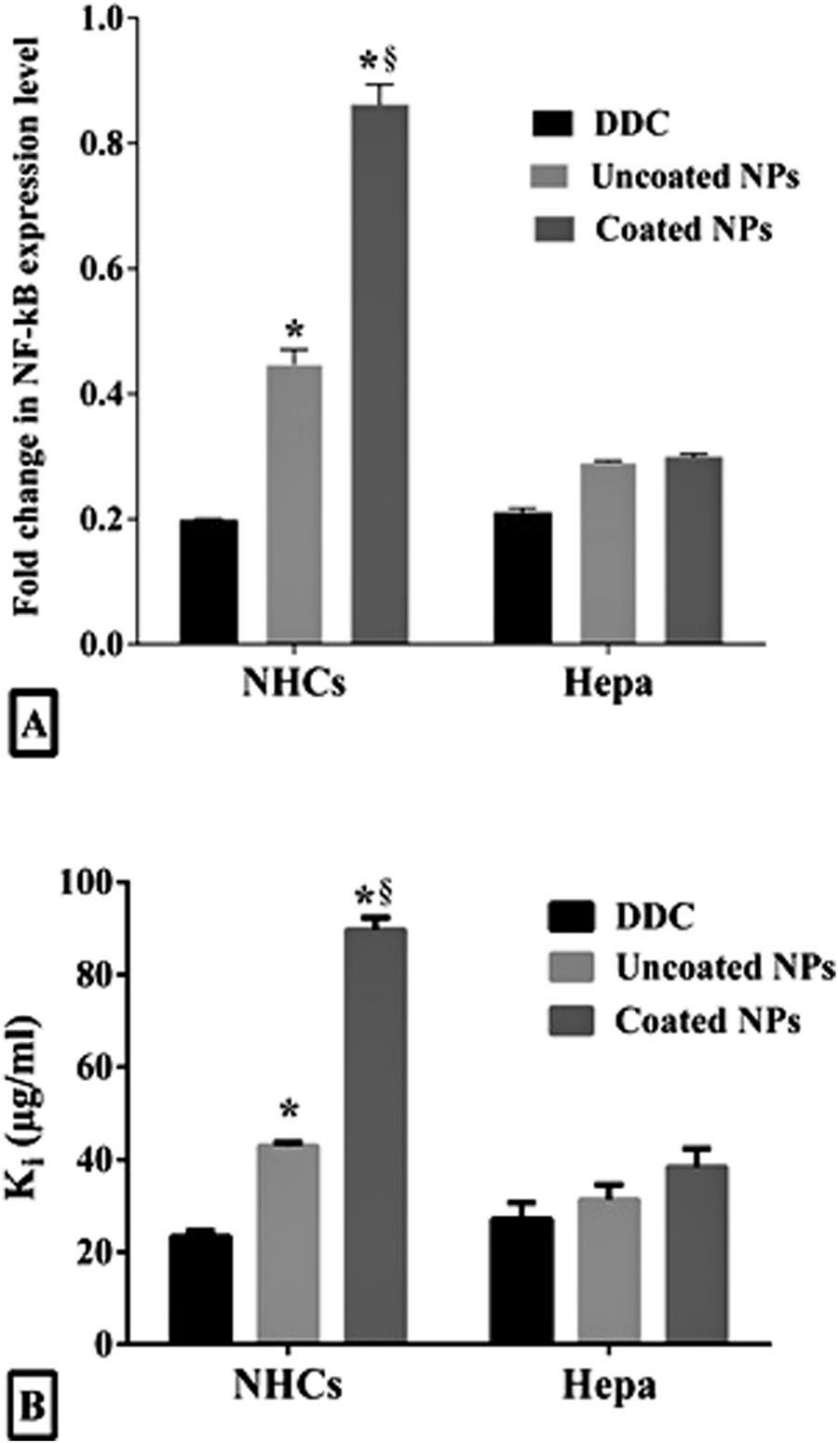
Downregulating effects of IC_50_ doses of DDC, uncoated NPs and coated NPs on (**A)** NF-κB expression and (**B)** ALDH1A1 activity (expressed as ki values) after incubation with NHCs and Hepa for 72 h (* and ^§^represent significance at p < 0.01 when compared to DDC-treated cells and uncoated NPs-treated cells, respectively).

As shown in Fig. 4A, the marked dowregulation (4.8, 3.4, 3.3 and 5.1 folds) of NF-κB expression was recorded in Hepa which were treated with IC_50_ of DDC, uncoated NPs and coated NPs as well as in DDC-exposed NHCs, respectively. Consistent with the selectivity of the studied NPs against Hepa, coated NPs (62.82 μg/ml) masked significantly (p < 0.001) the inhibitory effect of DDC on NF-κB expression in NHCs compared to uncoated NPs and free DDC.

Moreover, DDC inhibited ALDH1A1 activity (by 50%, k_i_) in normal and cancer hepatocytes at 23.29 ± 1.3 and 27.04 ± 3.6 μg/ml, respectively. Whereas, higher k_i_ (42.92 ± 0.81 μg/ml) was recorded for uncoated NPs in treated NHCs compared to DDC and it was decreased to 31.4 μg/ml in treated Hepa (Fig. 4B). More importantly, coated NPs were able to block inhibition of ALDH1A1 in NHCs by 50% up to 88 μg/ml DDC but 38.4 μg/ml DDC which loaded in the coated NPs was sufficient to inhibit ALDH1A1 in Hepa (Fig. 4B).

### The selective alteration in redox status of Hepa by the prepared NPs

**Figure 5:**
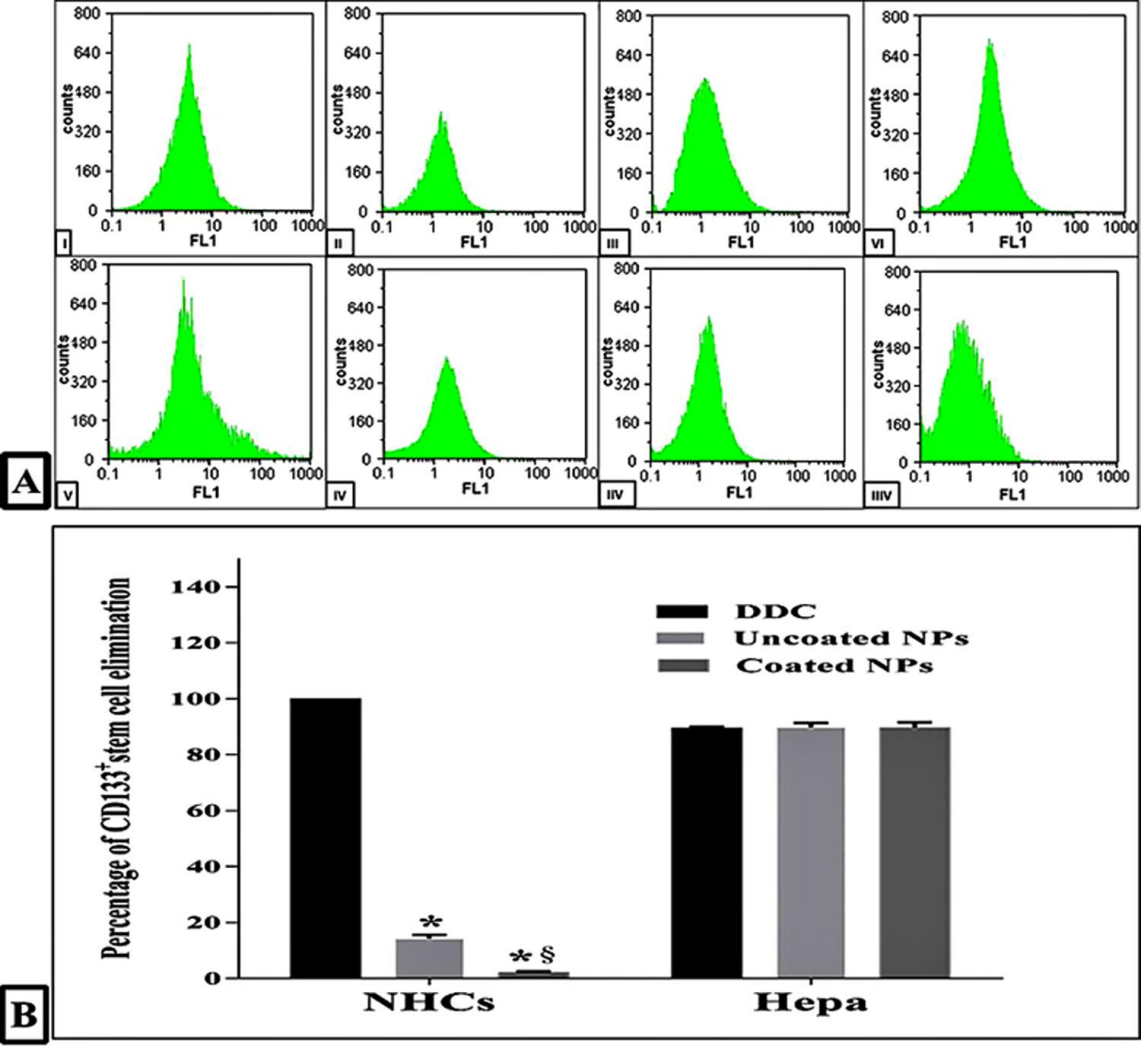
Flow cytometric analysis of relative DCF oxidation. (**A)** Flow charts of NHCs (I) after 72 h incubation with IC_50_ doses of DDC (II), uncoated NPs (III) and coated NPs (IV) as well as Hepa (V) after 72 h treatment with IC_50_ doses of DDC (VI), uncoated NPs (VII) and coated NPs (VIII). (**B)** The fold change in DCF fluorescence after treatment with DDC and its NPs relative to untreated NHCs and Hepa (* and ^§^represent significance at p < 0.01 when compared to DDC- treated cells and uncoated NPs-treated cells, respectively).

Figure 5A,B reveals that DDC, a well-known ALDH1 inhibitor and oxidizing agent, altered redox balance in both normal and cancer by increasing intercellular reactive species that caused oxidation of DCFH_2_ to DCF by ≈30%. Meanwhile, the coated NPs were able to protect NHCs from DDC-induced oxidation significantly than uncoated NPs-treated NHCs. As it was shown in the treated NHCs, the coated NPs halted elevation in DCFH_2_ oxidation by more than 87% compared to only 69% for uncoated NPs. On the other hand, both NPs modified the redox balance of Hepa by the same extent of DDC (>9 folds compared to untreated cells) and that was illustrated by a non-significant difference in the increasing rate of fluorescence intensity.

### The selective lethal effect of the studied NPs on hepatic CSCs

**Figure 6:**
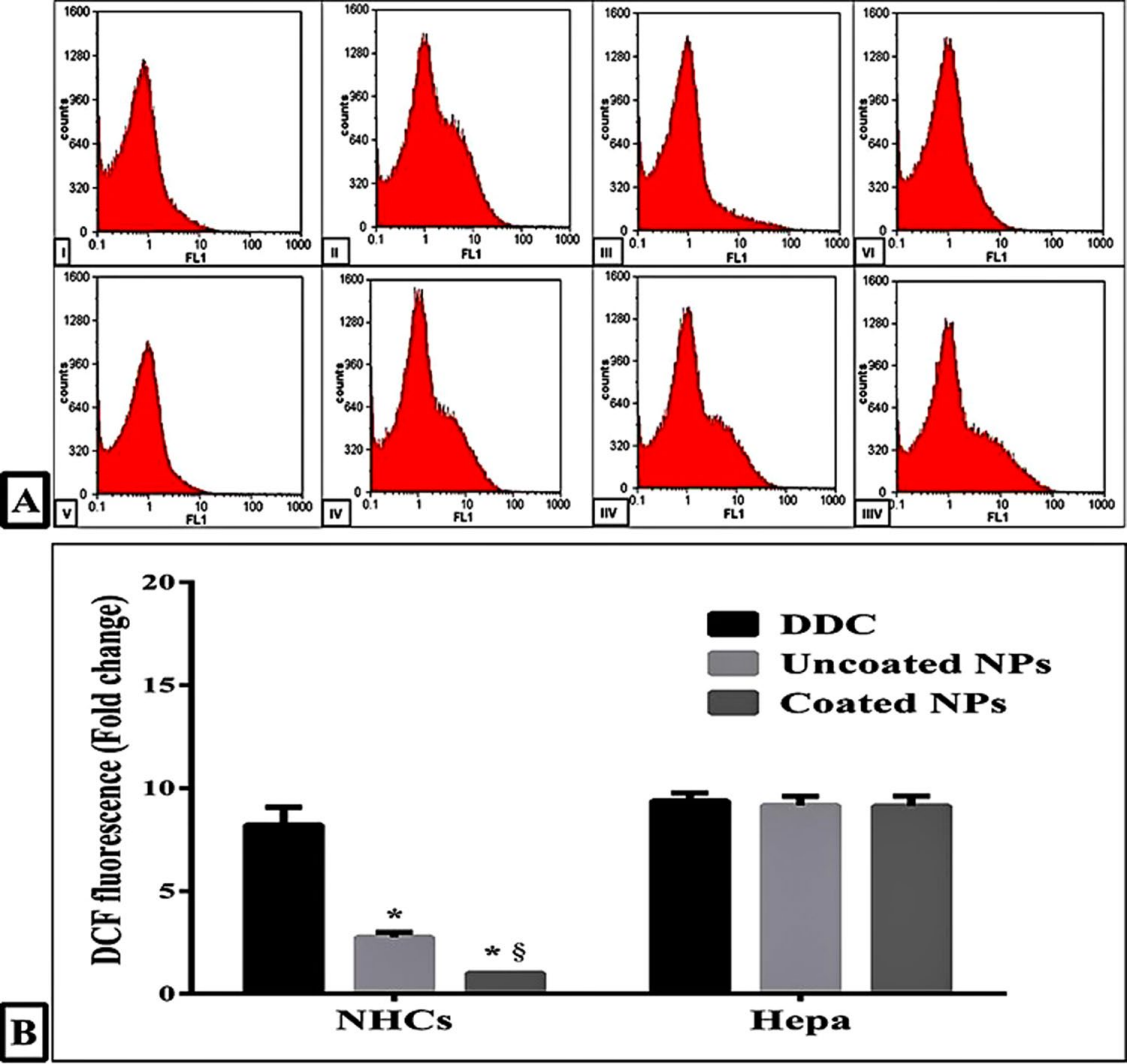
Flow cytometric analysis of CD133^+^ normal and cancer stem cells. (**A)** Flow charts of NHCs (I) after 72 h incubation with IC_50_ doses of DDC (II), uncoated NPs (III) and coated NPs (IV) as well as Hepa (V) after 72 h treatment with IC_50_ doses of DDC (VI), uncoated NPs (VII) and coated NPs (VIII). (**B)** The estimated elimination percentage of CD133^+^ normal and cancer stem cells after treatment (* and ^§^represent significance at p < 0.01 when compared to DDC-treated cells and uncoated NPs-treated cells, respectively).

Inhibition of ALDH1A1 and its consequence of oxidative stress result in collapsing stem cells. Consistent with the specific inhibitory effect of coated NPs on ALDH1A1 in Hepa, it eliminated CD133^+^ CSCs by 89.68 ± 2% and only 2.2 ± 0.3% for normal hepatic stem cells (Fig. 6A,B). Also, uncoated NPs exterminated 89.73 ± 1.6% of CSCs but more than 14% of normal stem cells were annihilated, so coated NPs was the highest selectivity to eradicate CSCs without causing a lethal effect on normal stem cells. In contrast, the percentage of elimination for CD133^+^ stem cells in both DDC-treated NHCs and Hepa was >89% (Fig. 6A,B).

### Targeted apoptotic effect of the studied NPs

**Figure 7:**
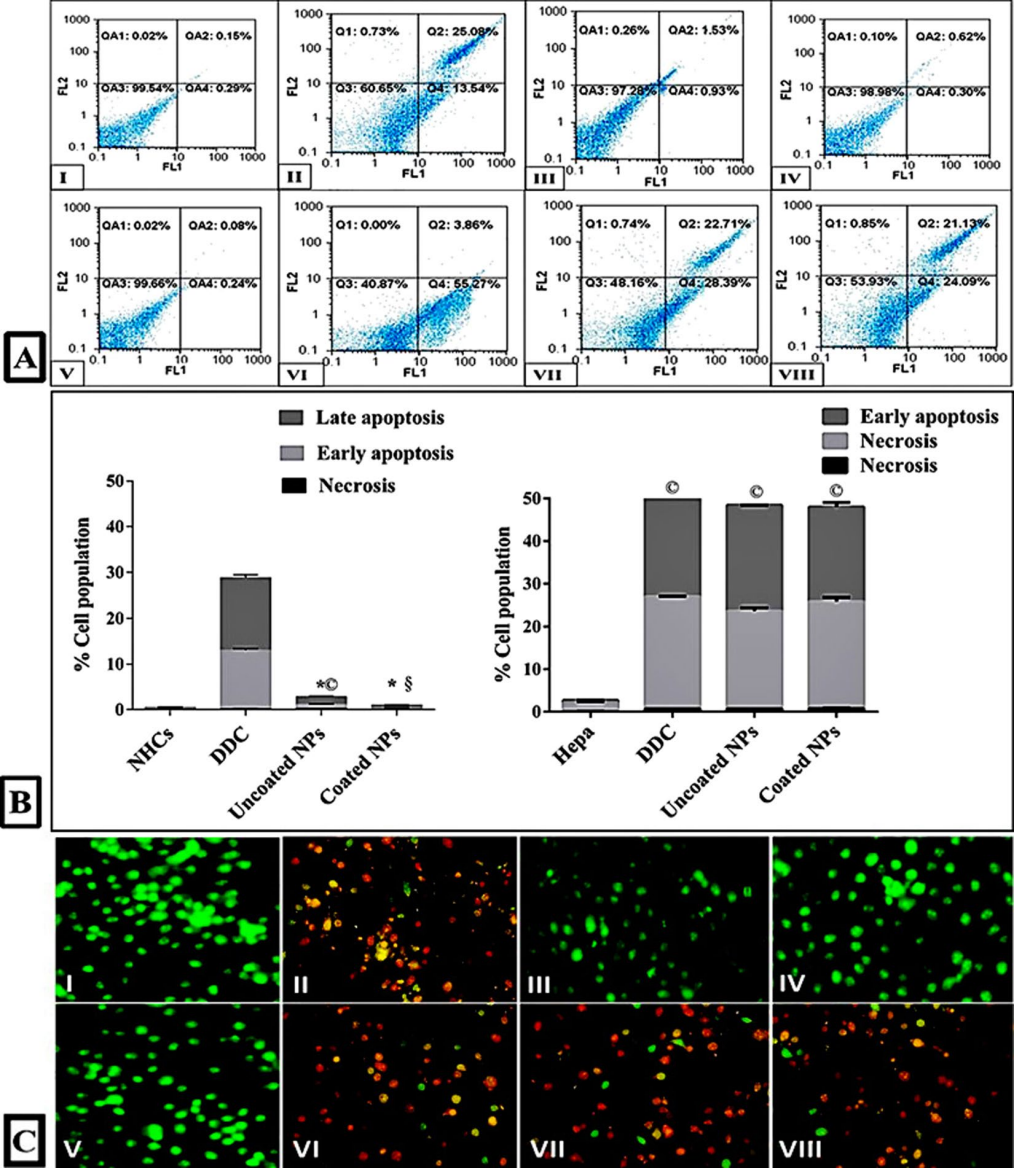
Selective apoptotic effect of IC_50_ doses of uncoated NPs and coated NPs in control with DDC. (**A)** Flow charts of annexin/PI-stained NHCs and Hepa before and after treatment. (**B)** The percentage of total apoptotic NHCs and Hepa before and after treatment. (^©,^* and ^§^represent significance at p < 0.01 when compared to untreated control cells, DDC-treated cells and uncoated NPs-treated cells, respectively). (**C)** Fluorescence photomicrographs of NHCs (I) after 72 h treatment with DDC (II), uncoated NPs (III) and coated NPs (IV) as well as Hepa (V) after 72 h treatment with DDC (VI), uncoated NPs (VII) and coated NPs (VIII).

Figure 7A,B presents that Hepa were collapsed mostly by apoptosis-dependent death that was induced by above 47% in all treated Hepa with DDC and its NPs. More importantly, flow cytometric analysis disclosed the selectivity of DDC-loaded NPs for induction of apoptosis-dependent death only in treated Hepa. Therefore Fig. 7A,B corroborated the safety of uncoated and coated NPs toward NHCs, significantly coated NPs where total apoptosis percentages of treated NHCs were 2.56 ± 0.1% and 0.92 ± 0.05%, respectively.

Furthermore, AO-EB nuclear stains harmonize with the above data by revealing green fluorescence for nuclei of NPs-treated NHCs as a sign of viable cells as in untreated NHCs and untreated Hepa (Fig. 7C). While emission orange or red fluorescence is an indicator of apoptotic cells as that was exhaled by Hepa or NHCs were treated with DDC and NPs-treated Hepa (Fig. 7C).

**Figure 8:**
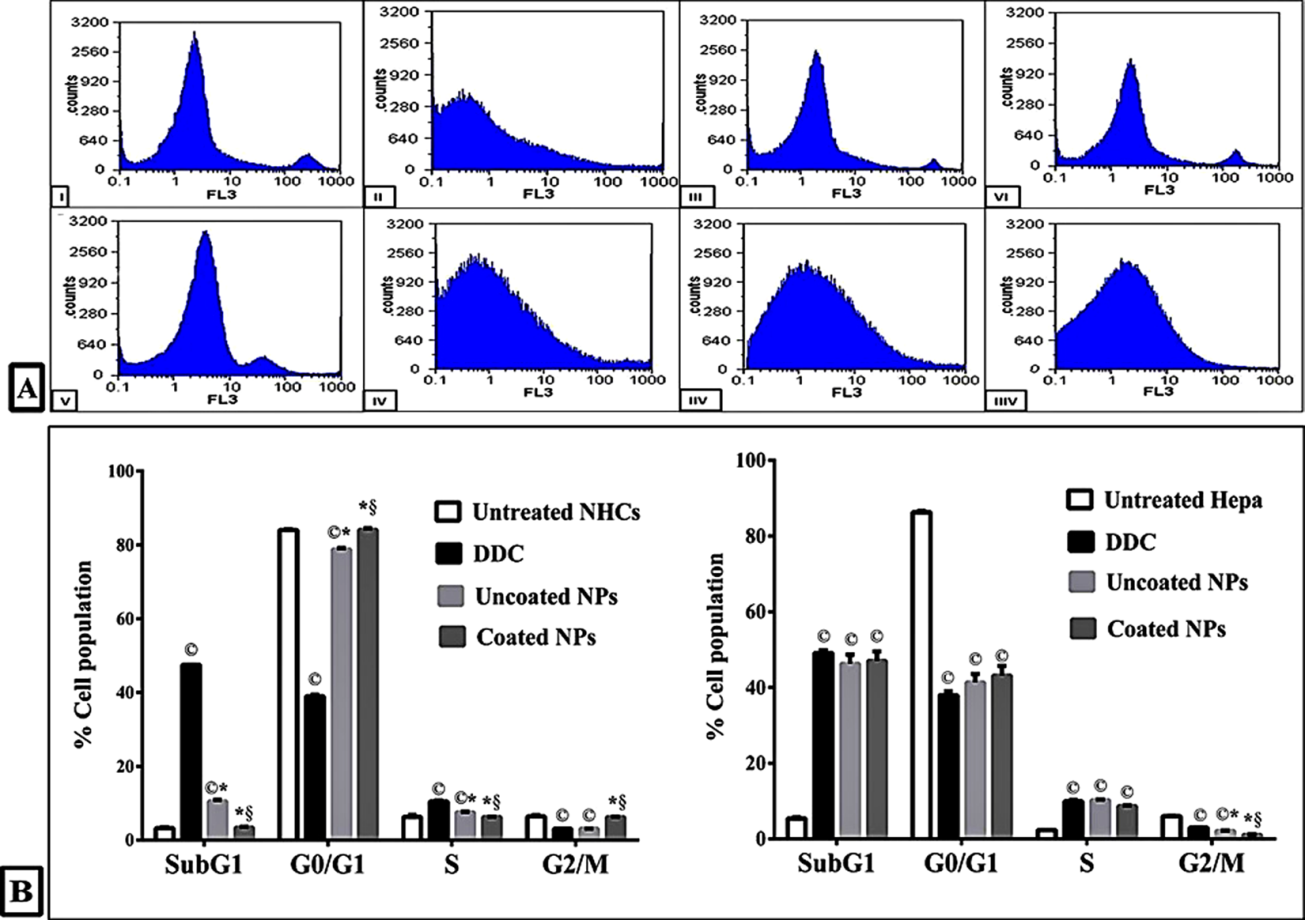
Alteration in cell cycle phases of treated NHCs and Hepa. (**A)** Flow charts of cell cycle diagrams of NHCs (I) after 72 h treatment with IC_50_ doses of DDC (II), uncoated NPs (III) and coated NPs (IV) as well as Hepa (V) after 72 h treatment with IC_50_ doses of DDC (VI), uncoated NPs (VII) and coated NPs (VIII). (**B)** Quantitative distribution percentages of cell cycle phases for untreated and treated NHCs and Hepa (^©,^* and ^§^represent significance at p < 0.01 when compared to untreated control cells, DDC-treated cells and uncoated NPs-treated cells, respectively).

Moreover, DDC and its NPs arrested cell cycle of Hepa at G1/S phase that was illustrated by the marked accumulation of treated Hepa in S phase to be more than 8.5% in comparison with 2.3 ± 0.04% of untreated cells. This accumulation was accompanied by the significant decrease in the percentage of cell population at G0/G1 phase (less than 44%) and statistically increased in the sub G1 phase from 5.39 ± 0.3% to > 46% (Fig. 8A,B). Consistency with above data (Figs 3–7), DDC induced cell cycle arrest at G1/S phase (10.5 ± 0.3%) with the highest percentage of NHCs population at sub G1 phase (47.5 ± 0.07%). Meanwhile, the coated DDC-loaded NPs were able to mask the apoptotic effect of DDC toward NHCs, more than uncoated NPs (p < 0.01), as it was clarified by non-significant differences between untreated NHCs and coated NPs-treated NHCs in all cell cycle phases (Fig. 8A,B).

## Discussion

One of the major challenges in the usage of anticancer drugs is toxicity against healthy tissue, so nanomedicine is critically needed for selectively targeting of tumor cells of liver. Previous evidences have emerged that targeting of CSCs is the effective therapeutic approach in liver cancer^3,4^. Therefore, DDC, as an inhibitor of ALDH1A1 (a key enzyme in the stemness of hepatic CSCs)^9^, was selected in this current study to load in chitosan for preparing uncoated NPs and BSA-coated NPs. Zeng *et al*.^32^ found that high distribution and absorption of labeled chitosan in liver tissue of mice after oral administration, indicating that absorbed chitosan had some accumulation in hepatocytes. Thus chitosan was the best choice as DDC carrier for targeting liver cancer cells.

The zeta potential results of the current study revealed that the uncoated NPs carried a positive charge (+45.4 mv) which became a negative charge (−19 mv) after coating with anionic protein (BSA). Hence, albumin which coated chitosan-based NPs with negative charges gets these coated NPs in repulsion with cell membranes of both normal and cancer cells. The detachment of albumin layer from these coated NPs only occurred in a slightly acidic pH, because of acidic hydrolysis of the electrostatic bond between BSA and chitosan, as it was confirmed by their zeta potential (+28.5 mv), as result of acidic hydrolysis the electrostatic bond between BSA and chitosan. Meanwhile, these coated NPs still possessed their negative charge in neutral pH which confirmed the presence of coating layer^27^. This indicated that the positive charge (+28.5 mv) of DDC-loaded NPs was reversed only in an acidic environment. Accordingly, these NPs are more attractive to cancer cell membranes than normal cell membranes. Thus, these reversed charge (coated) NPs had a highly selective attack on cancer cells whose slightly acidic microenvironment compared to a neutral microenvironment of normal cells. As it was affirmed by the pH-dependent DDC release from these albumin-coated NPs was higher than that from the uncoated NPs. This pH-dependent charge reversibility of the coated NPs interpreted the highest liberation rate (>80%) of DDC in acidic pH and the controlled release (≈20%) of DDC in neutral pH. In contrast to the uncontrolled DDC release (>40%) as free drug and from uncoated NPs that was observed in both neutral and acidic pHs. Based on the fact that electrostatic interactions between chitosan and albumin hydrolyze only in acidic pH (i.e. tumor microenvironment) and that indicates these switchable charge coated NPs reveal a protecting shell toward normal cells and become sticky to tumor cells^25,27^.

Accordingly, herein, uncoated NPs and especially coated NPs exhibited the highest safety toward NHCs compared to free drug DDC without observing a considerable difference between their anticancer activities on Hepa. Consequently, the coated DDC-loaded NPs possessed the highest SI between normal and cancerous liver cells. However, previous studies reported *in vitro* anticancer effect of DDC against several cancer cell lines and its *in vivo* enhancing potency for the anticancer effect of adriamycin NPs against liver cancer^33^. Several preclinical studies have provided evidence that DDC has toxicity toward healthy cells^16,34–36^. Our recent study proved that loading of the oxidized form of DDC (disulfiram) into albumin-coated chitosan NPs masked toxicity of disulfiram to normal colon cells and increased its selectivity towards colon cancer cells^27^.

The extreme toxicity of DDC mainly related to its potential for inhibiting NF-κB expression^37–40^ and ALDH1A1 activity^9,10^. In cancer cells, NF-κB expression is upregulated in order to transcript diverse target genes that mediate carcinogenesis, angiogenesis and metastasis as well as chemoresistance and proliferation of CSCs. Therefore, the inhibition of NF-κB expression presents a potential cancer treatment strategy^41,42^. DDC has been described as an inhibitor of NF-κB as a mechanism for decreasing expression of oncogenes like Bcl2^37–40^. Concomitantly, DDC is an irreversible inhibitor of ALDH1 by carbamylation of its catalytic Cys302 residue.^9,10^ ALDH1 protects normal and cancerous stem cells against oxidative stress (particularly reactive aldehydes), so inhibition of ALDH1A1 by DDC lead to severe alteration in cellular redox state and then cells lose their resistance to oxidative damage^7,43^. Ma *et al*.^6^ found that ALDH expression and activity in liver cancer cell lines were positively correlated with CD133 expression. In addition, CD133^+^ALDH^+^ cells were observed to be significantly more tumorigenic than CD133^+^ALDH^**−**^ cells or CD133^**−**^ALDH^+^ cells, both *in vitro* and *in vivo*^6^.

Obviously, in this current study, coated NPs were able to inhibit NF-κB expression and ALDH1A1 as well as alter redox balance selectively in cancer cells. Moreover, coated NPs did further possess the highest selectivity between CD133^+^CSCs of Hepa and CD133^+^ normal stem cells of NHCs. The inhibiting ALDH1A1 activity and high eliminating the percentage of CSCs lead to missing unique features of cancer cells such as uncontrolled proliferation, self-renewal and drug resistance^5,44^. All of these led to more than 47% of Hepa collapsed mostly by apoptosis-dependent death in all treated Hepa with DDC and its NPs that was assured by the highest percentage of annexin-stained cells, orange-red fluorescence stained nuclei and cell population in sub G1 phase. Induction of apoptosis coupled with cell cycle arrest is considered as only an advantageous strategy for cancer therapy^38^.

The maximum effects on cancer cells with minimal side effects on normal cells at low concentrations of drugs represent a great efficiency for cancer therapy. Previous *in vitro* and *in vivo* studies have been reported that DDC induced apoptosis in cancer cells (pancreatic cancer and lymphoma) as well as in normal cells (fibroblast)^17,39,40^. Meanwhile, a recent study confirmed that coated NPs which were loaded with the oxidized form of DDC stimulated apoptosis only in colon cancer cells rather than in normal colonic epithelial cells^25^. Also, this existing study succeeded to block cytotoxicity of DDC to NHCs and boosting its cancer selectivity by exploiting the charge switchable (coated) NPs. Thus DDC loaded BSA-coated NPs present a promising safe anticancer drug based on CSC-targeted therapy.

Hence, these charge switchable (DDC-loaded) NPs could be applied for selective targeting cancer cells, based on pH difference between normal and cancerous cells, with minimal effect on normal tissues. This novel study is considered a critical preliminary step for future preclinical studies of several cancer types.

## Materials and Methods

### Materials and reagents

William’s E medium, fetal bovine serum (FBS), trypsin-EDTA, L-glutamine and RPMI medium were obtained from Lonza (Belgium, USA). Collagenase I, sodium tripolyphosphate (TPP), BSA, Ellman’s reagent, pyrazole, NAD^+^, propionaldehyde, 2′,7′-dichlorodihydrofluorescein diacetate (DCFH_2_-DA), Annexin V-biotin, propidium iodide (PI), streptavidine fluorescein, ethidium bromide and acridine orange were purchased from Sigma (St Louis, USA). Chitosan and sodium DDC were from Acros Organics (New Jersey, USA). Gene JET RNA purification kit, cDNA synthesis kit and SYBR-green PCR assay kit were from Thermo Fisher Scientific (Waltham, Massachusetts, USA). Fluorescein isothiocyanate (FITC)-conjugated anti-mouse CD133 was obtained from BioLegend (San Diego, USA).

### Isolation and cultivation of normal hepatocytes

Black mice (C57 BL/6) were obtained from MISR University for Science and Technology with animal welfare assurance no. A5865–01. All applicable international, national, and/or institutional guidelines for the use of animals were followed. The use of experimental mice follows the Research Ethical Committee (REC) which was published by the National Health and Medical Research Council policies and the recommendations of Ministry of Health and Population, High Committee of Medical Specialties, Egypt. This current research was granted permission by Medical Biotechnology Department (SRAT-City).

Normal hepatocytes (NHCs) were isolated from C57 BL/6 mice according to the method of Whitehead and Robinson (2009)^45^ with some modifications. After mincing liver tissue, it was incubated with collagenase I (3 U/ml) with gentle shaking for 30 min and centrifuged at 1000 rpm for 5 min. Then cell pellet was suspended in William’s E medium supplemented with 10% FBS and incubated in 5% CO_2_ incubator at 37 °C. After 90% cell confluent, NHCs were passaged with trypsin-EDTA.

### Cell line and culture conditions

Black mouse C57 hepatoma (Hepa 1–6) cell line (Hepa) was obtained from American Type Culture Collection (ATCC, USA) and cultured in RPMI medium supplemented with 10% FBS and 2 mM L-glutamine in 6% CO_2_ incubator (to keep cellular pH nearly 6.5 and to mimic *in vivo* tumor microenvironment) at 37 °C.

### Preparation of uncoated DDC-loaded NPs and albumin-coated DDC-loaded NPs

Chitosan NPs were prepared by the process of ionic gelation method according to Anitha *et al*.^46^ with some modifications^25^. Chitosan (MW 100,000–300,000) was dissolved in 1% acetic acid with continuous stirring and pH was adjusted to be 4.7 with 1 M NaOH. Then 0.1% sodium DDC was added dropwise over 30 min to the chitosan solution (0.2%). The NPs were formed spontaneously upon the addition of cross-linking agent (0.4% TPP). The uncoated NPs were obtained by centrifugation twice at 15000 rpm for 30 min at 4 °C. One part of these NPs was suspended in PBS for coating with 2.5 mg BSA. After centrifugation, the coated and uncoated NPs were resuspended in water and then freeze-dried before further use or analysis.

### Characterization of the prepared DDC-loaded NPs

The percentage of loaded and encapsulated DDC was estimated after centrifugation of uncoated NPs to quantify the amount of unloaded DDC in the supernatant. Then 250 μl of supernatant or serial concentrations of DDC was mixed with equal volume of 0.5 M phosphate buffer pH 8 and 500 μl of 0.4 mg/ml of Ellman’s reagent^47^. After 3 h, the absorbance was measured at 450 nm using a spectrophotometer (BMG LabTech, Germany).

The average size and zeta potential of the uncoated and coated DDC-loaded NPs (at pH 7.4 and 6.5) were determined using dynamic light scattering method (Malvern ZEN3600, England).

Shape and surface morphology of uncoated and coated NPs were determined by scanning electron microscopy (JEOL, model JSM-6460LV, Japan) at an acceleration voltage of 10 kV.

### Release kinetics of DDC and DDC-loaded NPs in pH of normal cells and cancer cells

Diethyldithio-carbamate and the freeze-dried uncoated and coated DDC-loaded NPs were immersed (5 mg/mL) in PBS buffer pH 7.4 and PBS buffer pH 6.5. Then DDC and NPs were dialyzed (molecular weight cut-off 12 kDa) against PBS pH 7.4 and 6.5 under stirring in a shaker bath (100 rpm) at 37 °C. The amount of released DDC was determined using Ellman’s reagent, as described above, after 2, 4, 6, 8, 10, 12, 24, 48 and 72 h.

### Cytotoxicity of DDC and its prepared NPs against hepatic normal and cancer cells

Serial concentrations of DDC and NPs were incubated with NHCs and Hepa for 6, 12, 24, 48 and 72 h in 5% and 6% CO_2_ incubators, respectively. The percentages of NHCs viability and growth inhibition of Hepa were determined using MTT assay^48^. The doses that cause 50% cell viability or toxicity (IC_50_) values of DDC and the prepared NPs for normal cells and cancer cells, respectively, were estimated via GraphPad Instat software. Also, the selectivity indexes of DDC and the prepared NPs toward Hepa were calculated. Furthermore, morphological changes of treated normal and cancer cells were investigated using phase contrast inverted microscope (Olympus, Japan) with digital image analysis system.

### Quantitative detection of DDC, uncoated NPs and coated NPs effect on the expression of NF-κB in normal and cancer stem cells

Murine NHCs and Hepa were incubated with IC_50_ doses (μg/ml) of DDC, uncoated DDC- and coated DDC-loaded NPs for 72 h in 5% and 6% CO2 incubators, respectively at 37 °C. Total RNAs of untreated and treated NHCs and Hepa were extracted and cDNA of each sample was synthesized from mRNA by reverse transcriptase-PCR. For real time PCR, cDNA was amplified with the use of a SYBR-green PCR assay kit. Specific primer sequences (Forward/Reverse) were 5′-TGCCCGTGTTGTGGTAACCTTGG-3′/5′-CGTGAGAGAGTTTTGTCCGCCCTT-3′ for nuclear factor kappa B (NF-κB) gene. Primer sequences (Forward/Reverse) for β-actin (Forward/Reverse) primers were 5′-AGAGGGAAATCGTGCGTGAC-3′/5′-CAATAGTGATGACCTGGCCGT-3′. The QPCR program was applied as one cycle of enzyme activation at 95 °C for 15 min and followed by 40 cycles of denaturation at 95 °C for 15 sec, annealing at 60 °C for 1 min and extension at 72 °C for 30 sec. The 2^−ΔΔCT^ equation was used to quantify a change in NF-κB expression.

### Determination the inhibitory effect of DDC, uncoated NPs and coated NPs on ALDH1A1 activity in normal and cancer cells

The lysates of NHCs and Hepa were freshly used for measurement of ALDH1A1 activity, after 72 h incubation with serial concentrations of DDC- and its loaded NPs. Briefly, 600 μl cell lysate was mixed with sodium pyrophosphate buffer (0.09 M, pH 8.5), 1 mM pyrazole, 5 mM NAD^+^ and 5 mM propionaldehyde (substrate). The rate of change in absorbance at 340 nm was measured over 3 min according to Marselos *et al*.^49^. The ALDH1A1 activity was calculated as nmoles NADH produced/min/mg protein and K_i_ was expressed as the concentration that causes 50% inhibition for ALDH1A1 activity.

### Flow cytometric assessment of lethal effect of DDC and DDC-loaded NPs on normal and cancer stem cells

The untreated and treated cells were trypsinized, suspended in PBS and fixed in 4% formaldehyde for 10 min. Then NHCs and Hepa were stained with 20 μl of FITC-conjugated anti-mouse CD133 for 30 min in the dark. These stained cells were analyzed on FACS (BD Biosciences, USA) at FITC signal detector; FL1. Then the decrease in percentages of CD133+ stem cells before and after treatment was quantified to detect toxicity effect of DDC and NPs on stem cells populations of hepatic normal and cancer cells.

### Determination the redox alteration in treated normal and cancer cells

Briefly, untreated and treated normal and cancer cells were incubated with 5 μM of DCFH_2_-DA in the dark for 30 min at 37 °C. After that, cells were trypsinized and suspended in a fresh phosphate buffer saline. The percentage of fluorescence DCF product which generates via the deacetylation and then oxidation of DCFH_2_ by intercellular reactive oxygen species was analyzed by flow cytometer with excitation and emission settings at 488 nm and 530 nm, respectively.

### Assessment of the selective cancer cell death by flow cytometry and fluorescence microscopy

Briefly, untreated and treated cells were trypsinized and incubated with 5 µl annexin V- biotin and 5 µl PI for 20 min in dark. After staining, cells were fixed with 2% formaldehyde for 15 min and incubated with 5 µg/ml of streptavidine-fluorescein for 15 min. The percentages of apoptotic and necrotic cells were detected by flow cytometry (Ex = 488 nm; Em = 530 nm) using Annexin V and PI double staining (FITC signal detector; FL1 and the phycoerythrin emission signal detector; FL2, respectively).

For fluorescence microscopy, cells were stained with 1 μl of 100 μg/ml ethidium bromide and 100 μg/ml acridine orange and then investigated under the fluorescence phase contrast microscope (Olympus, Japan).

### Cell cycle analysis

The untreated and treated cells were trypsinized and fixed in 70% cold ethanol at 4 °C for 10 min. After centrifugation, cells were resuspended in PBS containing 5 μg/ml RNase A. After an hour, cells were incubated with PI staining buffer for 15 min and then the percentages of different cell cycle phases (sub G1, G1, S and G2/M) were analyzed using flow cytometry (Partec, Germany) according to the method of Jass *et al*.^50^.

### Statistical analysis

All values (n = 6) were expressed as mean ± standard error of the mean (SEM). Statistical significance (p values < 0.01) was calculated by the multiple comparisons one-way analysis of variance (ANOVA) with post-hoc Tukey’s test using SPSS16 software program.

### Availability of data and materials

All data generated during this study is included in this published article.
